# Supplementary material for: Can We Estimate Functionality of Soil Microbial Communities from Structure-Derived Predictions? A Reality Test in Agricultural Soils
Source: Microbiol Spectr. 2021 Aug 4;9(1):10.1128/spectrum.00278-21. doi: 10.1128/spectrum.00278-21 (PMC8552701; doi:10.1128/spectrum.00278-21)
Supplement: SUPPLEMENTAL FILE 1 — Supplemental material. Download SPECTRUM00278-21_Supp_1_seq5.pdf, PDF file, 2.1 MB [file spectrum00278-21_supp_1_seq5.pdf]

Can we estimate functionality of soil microbial communities from structure-derived predictions? A reality test in agricultural soils

Claudia Breitkreuz[a]#, Anna Heintz-Buschart[b,a]#, François Buscot[a,b], Sara Fareed Mohamed Wahdan[a], Mika Tarkka[a,b], Thomas Reitz[a,b]

[a]Department of Soil Ecology, UFZ – Helmholtz Centre for Environmental Research, Halle/Saale, Germany

[b]German Centre for Integrative Biodiversity Research (iDiv) Halle-Jena-Leipzig, Leipzig, Germany

Supplementary Tables

Table S 1 Correlations between functional gene abundances predicted by Tax4Fun and PanFP and measured enzyme activities of β-glucosidases, xylosidases, N-acetylglucosaminidases (chitinases), acid phosphatases and cellobiohydrolases (cellulases). The correlation coefficients (Spearman's rho) and respective significance levels were calculated for wheat and barley rhizospheres for combined and separated growth phases. Significant impacts according to ANOVA are indicated by italic p values and significance levels according to Spearman rank correlation test are given as follows: <0.001(***), <0.01(**), <0.05(*) and p<0.1(.).

**Spearman rank correlation**		**Wheat**						**Barley**					
		**Total (n=40)**		**active (n=20)**		**mature (n=20)**		**Total (n=40)**		**active (n=20)**		**mature (n=20)**	
		**ρ**	**p value**	**ρ**	**p value**	**ρ**	**p value**	**ρ**	**p value**	**ρ**	**p value**	**ρ**	**p value**
**Tax4Fun vs. Enzymes**	Glucosidases	0.22	0.15	0.35	0.12	0.18	0.44	_0.44_	_0.003**_	_0.55_	_0.01*_	_0.41_	_0.06._
	Xylosidases	_0.45_	_0.003**_	_0.41_	_0.06._	_0.44_	_0.04*_	_0.41_	_0.008**_	_0.57_	_0.008**_	0.16	0.49
	Chitinases	0.19	0.23	0.32	0.16	0.009	0.97	_0.41_	_0.007**_	_0.40_	_0.08._	_0.39_	_0.08._
	Phosphatases	_0.30_	_0.05._	0.17	0.46	_0.50_	_0.02*_	0.08	0.58	_0.59_	_0.006**_	0.36	0.11
	Cellulases	0.17	0.28	0.15	0.51	0.28	0.22	_0.33_	_0.03*_	_0.38_	_0.09._	_0.46_	_0.04*_
**PanFP vs. Enzymes**	Glucosidases	0.23	0.13	0.34	0.14	0.29	0.20	_0.29_	_0.06._	_0.51_	_0.02*_	_0.46_	_0.04*_
	Xylosidases	_0.45_	_0.003**_	0.34	0.13	_0.53_	_0.01*_	_0.33_	_0.03*_	_0.42_	_0.06._	0.24	0.29
	Chitinases	0.20	0.21	0.18	0.43	0.09	0.67	_0.41_	_0.007**_	_0.38_	_0.09._	_0.38_	_0.09._
	Phosphatases	_0.27_	_0.08._	0.17	0.44	_0.41_	_0.07._	0.14	0.36	_0.60_	_0.005**_	_0.45_	_0.04*_
	Cellulases	0.05	0.71	0.33	0.15	-0.08	0.70	_0.27_	_0.08._	0.33	0.15	0.27	0.24

Table S 2 Drivers of Tax4Fun- and PanFP-predicted functional gene abundances. Abundances of genes encoding for β-glucosidases, xylosidases, N-acetylglucosaminidases (chitinases), acid phosphatases and cellobiohydrolases (cellulases) were tested against the factors farming system, growth phase, climate and interaction of farming system and growth phase. Significant impacts according to ANOVA are indicated by italic p values and significance levels are given as follows: p <0.001(***), p <0.01(**), p< 0.05(*) and p<0.1(.).

Predicted: Tax4Fun		Farming	Growth Phase	Climate	Growth Phase: Farming	Crop Species
**Wheat**	Glucosidases	0.12	0.15	0.18	0.25	
	Xylosidases	0.10	0.02*	0.13	0.27	
	Chitinases	0.09.	0.45	0.31	0.12	
	Phosphatases	0.10	0.001**	0.08.	0.47	
	Cellulases	0.20	0.003**	0.06.	0.42	
**Barley**	Glucosidases	0.01*	<0.001***	0.98	0.64	
	Xylosidases	0.02*	<0.001***	0.81	0.74	
	Chitinases	0.006**	0.29	0.62	0.25	
	Phosphatases	0.02*	<0.001***	0.97	0.84	
	Cellulases	0.02*	<0.001***	0.99	0.88	
**Wheat & Barley**	Glucosidases					<0.001***
	Xylosidases					<0.001***
	Chitinases					<0.001***
	Phosphatases					0.004**
	Cellulases					0.002**

Predicted: PanFP		Farming	Growth Phase	Climate	Growth Phase:Farming	Crop Species
**Wheat**	Glucosidases	0.10	0.01*	0.21	0.46	
	Xylosidases	0.09.	0.01*	0.22	0.41	
	Chitinases	0.13	0.07.	0.16	0.30	
	Phosphatases	0.10	0.01*	0.16	0.42	
	Cellulases	0.87	0.01*	0.94	0.80	
**Barley**	Glucosidases	0.05.	<0.001***	0.89	0.97	
	Xylosidases	0.10	<0.001***	0.96	0.91	
	Chitinases	0.08.	<0.001***	0.86	0.66	
	Phosphatases	0.06.	<0.001***	0.88	0.70	
	Cellulases	0.08.	<0.001***	0.88	0.81	
**Wheat & Barley**	Glucosidases					0.02*
	Xylosidases					0.09.
	Chitinases					0.003**
	Phosphatases					0.008**
	Cellulases					0.17

Table S3 Measured enzyme categories and respective 4-methylumbelliferon (MUF)-coupled substrates

Enzyme	Substrate
β-glucosidase (EC 3.2.1.21)	4-MUF-β-D-glucopyranoside
cellobiohydrolase (EC 3.2.1.91)	4-MUF-β-D-cellobioside
xylosidase (EC 3.2.1.37)	4-MUF-β-D-xylopyranoside
N-acetylglucosaminidase (EC 3.2.1.14)	4-MUF-N-acetyl-β-D-glucosaminide
acid phosphatase (EC 3.1.3)	4-MUF-phosphate

Table S4 Settings of PCRs used for next generation sequencing with Illumina and qPCR. Conditions for amplification of 16S rRNA gene region (PCR 1), index PCR (PCR 2) and qPCR are listed.

	**Step**	**Temperature (°C)**	**Time (min:sec)**
**PCR 1**			
	Initial denaturation	95	3:00
25 cycles	Denaturation	98	0:20
	Annealing	55	0:15
	Elongation	72	0:15
	Final extension	72	5:00
**PCR 2**			
	Initial denaturation	95	3:00
8 cycles	Denaturation	98	0:30
	Annealing	55	0:30
	Elongation	72	0:30
	Final extension	72	5:00
**qPCR**			
	Initial denaturation	95	5:00
40 cycles	Denaturation	95	0:10
	Annealing	55	0.30

Table S5 Enzyme categories extracted from output of Tax4Fun and PanFP prediction tools. The respective KEGG orthology (KO) identifiers and Enzyme Commission (EC) identifiers for each enzyme are given.

KO	Description (EC number)
**K05349**	beta-glucosidase [EC:3.2.1.21]
**K05350**	beta-glucosidase [EC:3.2.1.21]
**K01198**	1,4-beta-xylosidase [EC:3.2.1.37]
**K01205**	alpha-N-acetylglucosaminidase [EC:3.2.1.50]
**K01078**	acid phosphatase [EC:3.1.3.2]
**K03788**	acid phosphatase (class B) [EC:3.1.3.2]
**K09474**	acid phosphatase (class A) [EC:3.1.3.2]
**K01225**	1,4-beta-cellobiosidase [EC:3.2.1.91]

Table S6 Treatment-specific 16S rDNA gene abundances (given as µg DNA per g dry soil) in the rhizosphere. Treatment-specific means (± standard deviation in brackets) of DNA concentrations were calculated for both crops separately. Different small letters indicate significant differences between the treatments (ANOVA and Tukey HSD).

Crop/ Year	Growth Phase	Farming system†	Concentrations (µg DNA per g dry soil)
Wheat 2015	Active	CF	3.13 ab (1.22)
		OF	1.94 bc (1.20)
	Mature	CF	3.63 a (1.33)
		OF	3.26 ab (1.86)
Barley 2016	Active	CF	2.01 bc (0.90)
		OF	1.25 c (0.49)
	Mature	CF	3.28 ab (1.29)
		OF	2.69 abc (0.60)

† CF = conventional farming, OF = organic farming

Supplementary Figures

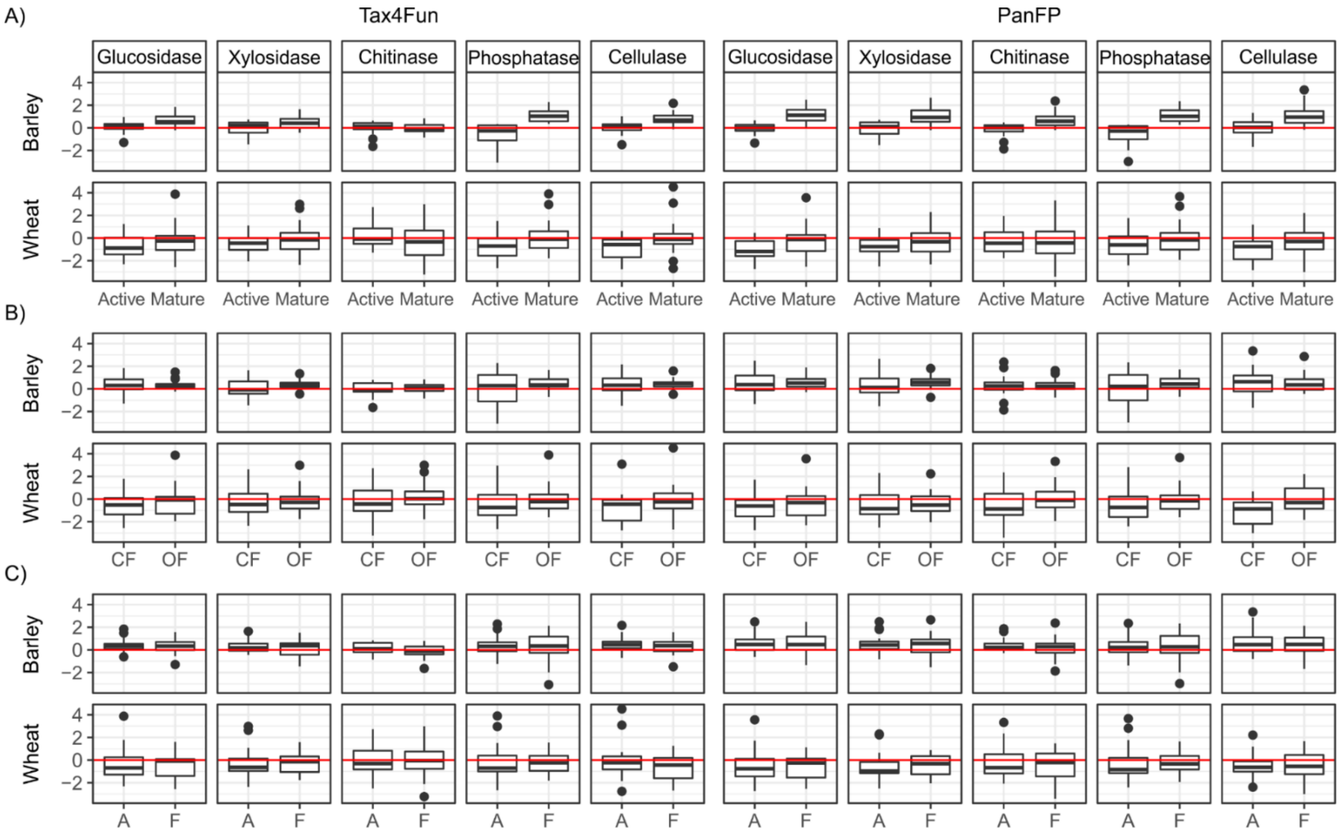

Figure S 1 Difference between measured and Tax4Fun and PanFP predicted z-transformed values. Differences are given for barley and wheat relative to A) active and mature growth phases, B) conventional (CF) and organic (OF) farming systems and C) ambient (A) and future (F) climate treatments. The red lines indicate zero deviations in levels of measured and predicted values, while positive values indicate overestimation and negative values underestimation of predictions compared to measured enzyme activities.

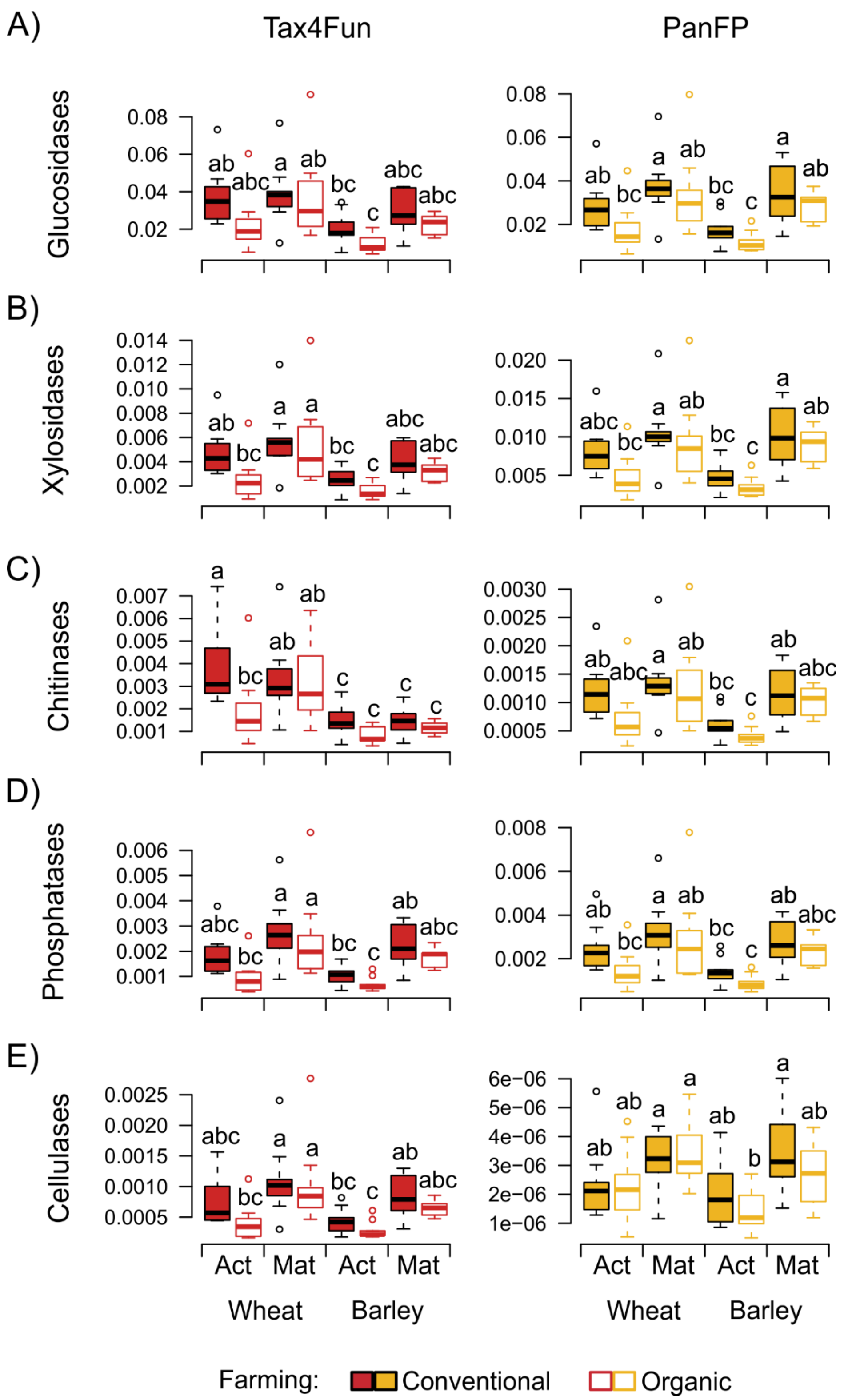

Figure S 2 Impacts of crop growth phase, farming system and crop species on predicted functional gene abundances. A) *β*-glucosidases, B) xylosidases, C) N-acetylglucosaminidases (chitinases), D) acid phosphatases, and E) cellobiohydrolases (cellulases). Predicted gene abundances by Tax4Fun (red) and PanFP (yellow) at the active (Act) and Mature (Mat) growth phases in conventional and organic farming soils are given. Different small letters within each subfigure indicate significant differences between the treatments (p<0.05) according to Tukey HSD.

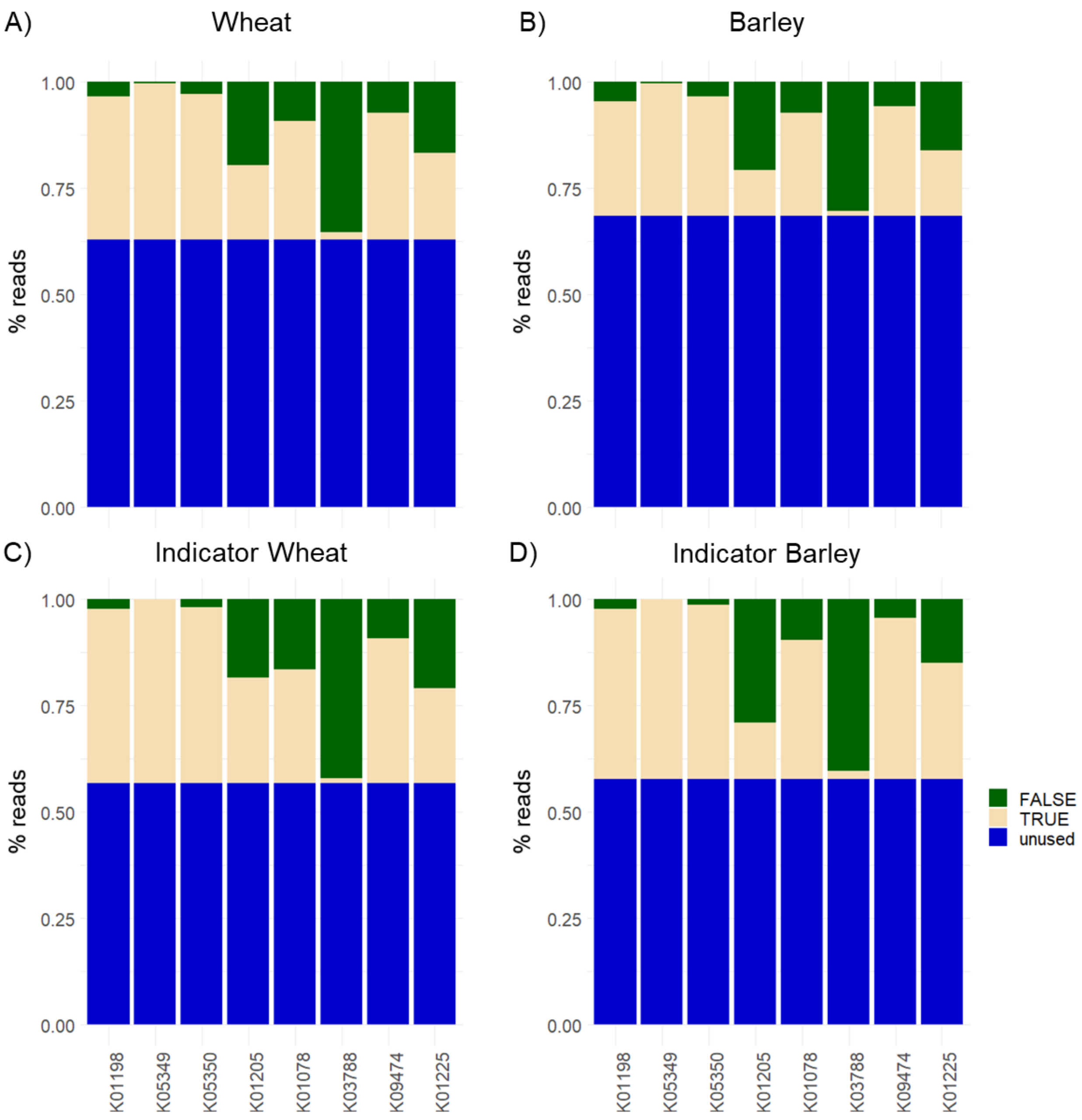

Figure S 3 Proportion of bacterial sequences used for functional predictions with Tax4Fun and containing the enzyme gens. Kegg orthology identifiers represent the five enzymatic classes referred to within this study (see Table S3 for more information on enzymes). Analysis was performed at OTU level, which were weighted by average number of reads in rhizospheres of A) wheat and B) barley, as well as by the respective indicator species abundance in C) wheat and D) barley. FALSE: gene not represented, TRUE: gene represented, unused: no information about presence or absence of gene.

Supplemental Material 1:

Variation in edaphic parameters according to experimental factors

Among the tested abiotic soil parameters, mineral nitrogen concentration (ANOVA, F = 62.74, p < 0.001) as well as total carbon (ANOVA, F = 11.52, p = 0.001) and total nitrogen content (ANOVA, F = 7.47, p = 0.008) in the soil were influenced by farming practice. Thereby, the concentrations were higher in conventional than in organic farming systems (Table SM 1). Moreover, mineral nitrogen concentration (ANOVA, F = 51.96, p < 0.001) and soil moisture (ANOVA, F = 1894.94, p < 0.001) differed significantly between the two growth phases. Mineral nitrogen concentration and soil moisture were higher at the mature than the active growth phase (Table SM 1). Measured soil moisture rather displays short-term changes due to rainfall events at sampling dates than overall climate treatment. An effect of the crop species in each year was found for total nitrogen content (ANOVA, F = 8.20, p = 0.006) and mineral nitrogen concentrations (ANOVA, F = 43.33, p < 0.001). For pH and P neither an effect of the farming system nor of crop species or growth phase could be detected.

Table SM 1 Abiotic soil parameters. Treatment-specific means (± standard deviation in brackets) are given for mineral nitrogen (N_{min}), total carbon (TC), total nitrogen (TN), pH, soil moisture, available phosphor (P_{av}). Different small letters within each column indicate significant differences between the treatments.

Crop/ Year	Growth Phase	Farming system†	N_{min} (mg/kg)	TC (%)	TN (%)	pH	Moisture (%)	P_{av} (mg/kg)
Wheat 2015	Active	CF	10.79 ab (5.15)	2.05 a (0.07)	0.17 a (0.01)	6.79 (0.50)	10.45 a (0.91)	82.4 (40.6)
		OF	4.35 def (0.89)	1.94 ab (0.07)	0.16 ab (0.01)	6.64 (0.56)	10.57 a (0.33)	73.4 (39.7)
	Mature	CF	13.45 a (2.31)	1.95 ab (0.20)	0.16 ab (0.02)	6.82 (0.52)	15.94 b (0.39)	83.1 (40.6)
		OF	7.49 bcd (1.27)	1.85 b (0.28)	0.14 b (0.03)	6.71 (0.59)	15.74 b (0.56)	72.4 (36.4)
Barley 2016	Active	CF	3.96 ef (1.02)	2.01 ab (0.09)	0.17 a (0.01)	6.79 (0.48)	9.85 a (0.59)	75.0 (37.3)
		OF	2.71 f (0.41)	1.92 ab (0.09)	0.16 ab (0.01)	6.66 (0.55)	10.58 a (0.75)	71.2 (40.9)
	Mature	CF	9.26 bc (2.78)	2.00 ab (0.07)	0.18 a (0.01)	6.81 (0.52)	16.08 b (0.73)	73.6 (36.9)

OF	6.53 cde (2.26)	1.86 ab (0.09)	0.16 ab (0.01)	6.64 (0.58)	15.79 b (0.68)	70.0 (37.7)

† CF = conventional farming, OF = organic farming

Supplemental Material 2

Effect of abiotic soil parameters on bacterial community structure and function

Plot-specific soil pH was found to be the most prominent edaphic factor shaping rhizosphere bacterial community composition (Figure SM 1, PERMANOVA, R^2=0.20, p<0.001), followed by soil moisture (PERMANOVA, R^2=0.16, p<0.001), available phosphate concentration (PERMANOVA, R^2=0.14, p<0.001), mineral nitrogen concentration (PERMANOVA, R^2=0.05, p=0.003) and total carbon content (PERMANOVA, R^2=0.05, p=0.003). No effect of total nitrogen content on community composition was found (PERMANOVA, R^2=0.02, p=0.33).

Contrary to the community composition, enzyme activities in the rhizosphere of wheat and barley were mainly driven by mineral nitrogen and TC (Table SM 1). While the effect of mineral nitrogen was mainly pronounced in the rhizosphere of wheat, the effect of TC was mainly driving enzyme activities in the barley rhizosphere (Table SM 1). TN influenced enzyme activities of glucosidases and cellulases in the rhizospheres of wheat and barley. Soil moisture had only effects on phosphatases in the barley rhizosphere and on chitinases in the wheat rhizosphere. Available phosphate concentration and pH did not affect enzyme activities (Table SM 1).

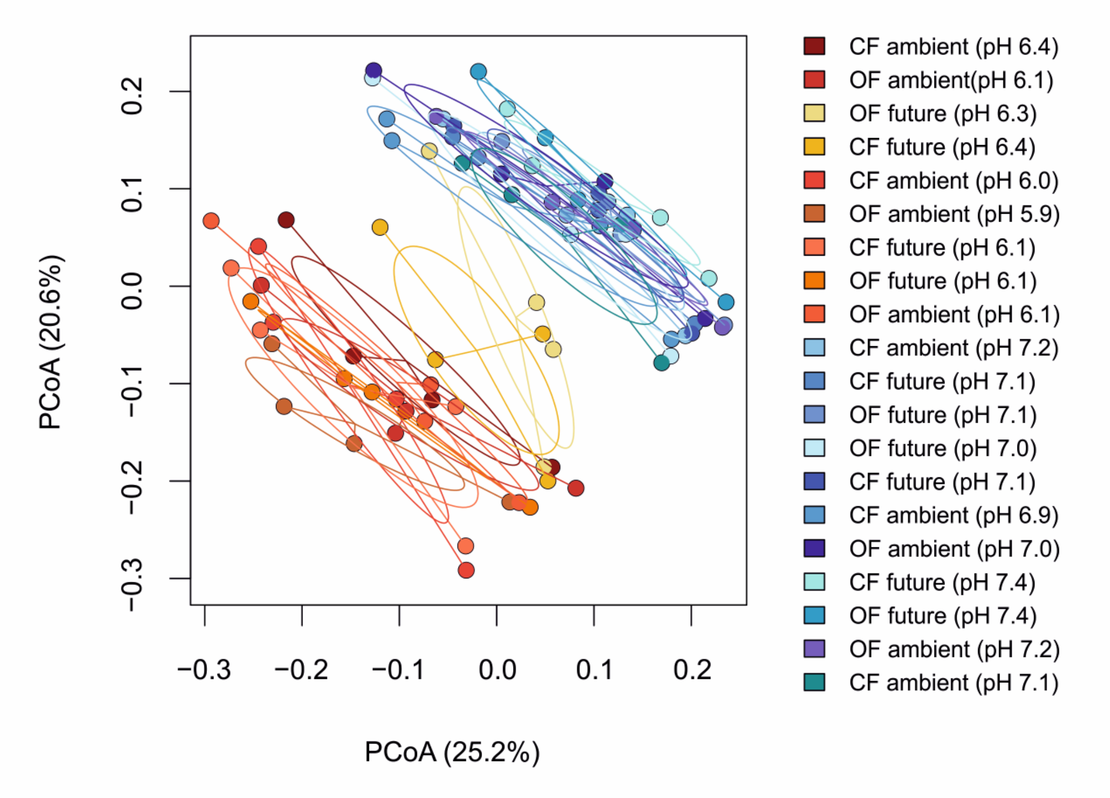

Figure SM 1 Comparison of the beta diversity of the rhizosphere bacterial communities between all plots (n = 80). Principal component analysis based on OTU abundances. Points are colored and connected according to origin of samples from the plots of the GCEF and respective treatments of farming - conventional farming (CF) and organic farming (OF) - and climate - ambient and future climate. Means of the respective pH values at the four time points sampled are given in brackets.

Table SM 1 Impact of abiotic soil parameters on rhizosphere enzyme activities. Activities of beta-glucosidases, xylosidases, N-acetylglucosaminidases (chitinases), acid phosphatases and cellobiohydrolases (cellulases) were tested against soil pH, soil moisture, mineral nitrogen (N_{min}), available phosphor (P), total carbon (TC) and total nitrogen (TN). Significant impacts according to ANOVA are indicated by italic p values and significance levels according to ANOVA are given as follows: p < 0.001(***), p < 0.01(**), p < 0.05(*) and p < 0.1(.).

**Measured**		**pH**	**Moisture**	**N_{min}**	**P**	**TC**	**TN**
	Glucosidases	0.50	0.60	*0.01**	0.20	*0.02**	*0.005***
	Xylosidases	0.35	*0.09.*	0.11	0.67	0.17	*0.05.*
**Wheat**	Chitinases	0.60	*0.01**	*0.002***	0.95	0.17	0.17
	Phosphatases	0.31	0.23	*0.005***	*0.09.*	0.11	*0.06.*
	Cellulases	0.53	0.80	*0.01**	0.28	*0.01**	*0.01**
	Glucosidases	0.36	0.44	0.44	0.40	*0.002***	*0.03**
	Xylosidases	0.46	0.40	0.18	0.43	*0.01**	0.16
**Barley**	Chitinases	0.58	0.51	0.21	0.92	*0.05.*	0.58
	Phosphatases	0.41	*0.001***	0.28	0.86	*0.08.*	0.92
	Cellulases	0.39	0.72	0.32	0.36	*<0.001****	*0.03**

Supplemental Material 3

Indicator species analysis

To identify OTUs that drove the observed separation according to experimental factors, indicator species analysis was performed (Figure SM 1). Over the four sampling times and in relation to the farming system treatment 950 and 564 indicator species were identified for wheat and barley, respectively. The phylum-level composition of the set of indicator species was comparable to the total community (Fisher test; Table SM 1 and SM 2). For barley, we found a clear separation between growth phases and across the two farming systems with 122 (21.6%) at active and 143 (25.4%) common indicator species at mature growth phases (Venn diagram, Figure SM 1). Only 24 (4.3%) and 14 (2.5%) indicator species were shared within CF and OF at different growth phases. In contrast, indicator species distribution for wheat showed a partially different pattern (Figure SM 1). While we observed 75 (7.9%) and 84 (8.8%) growth phase-specific shared indicator species, we identified 225 (23.7%) and 72 (7.8%) shared indicator species at different growth phases in CF and OF, respectively (Venn diagram, Figure SM 1).

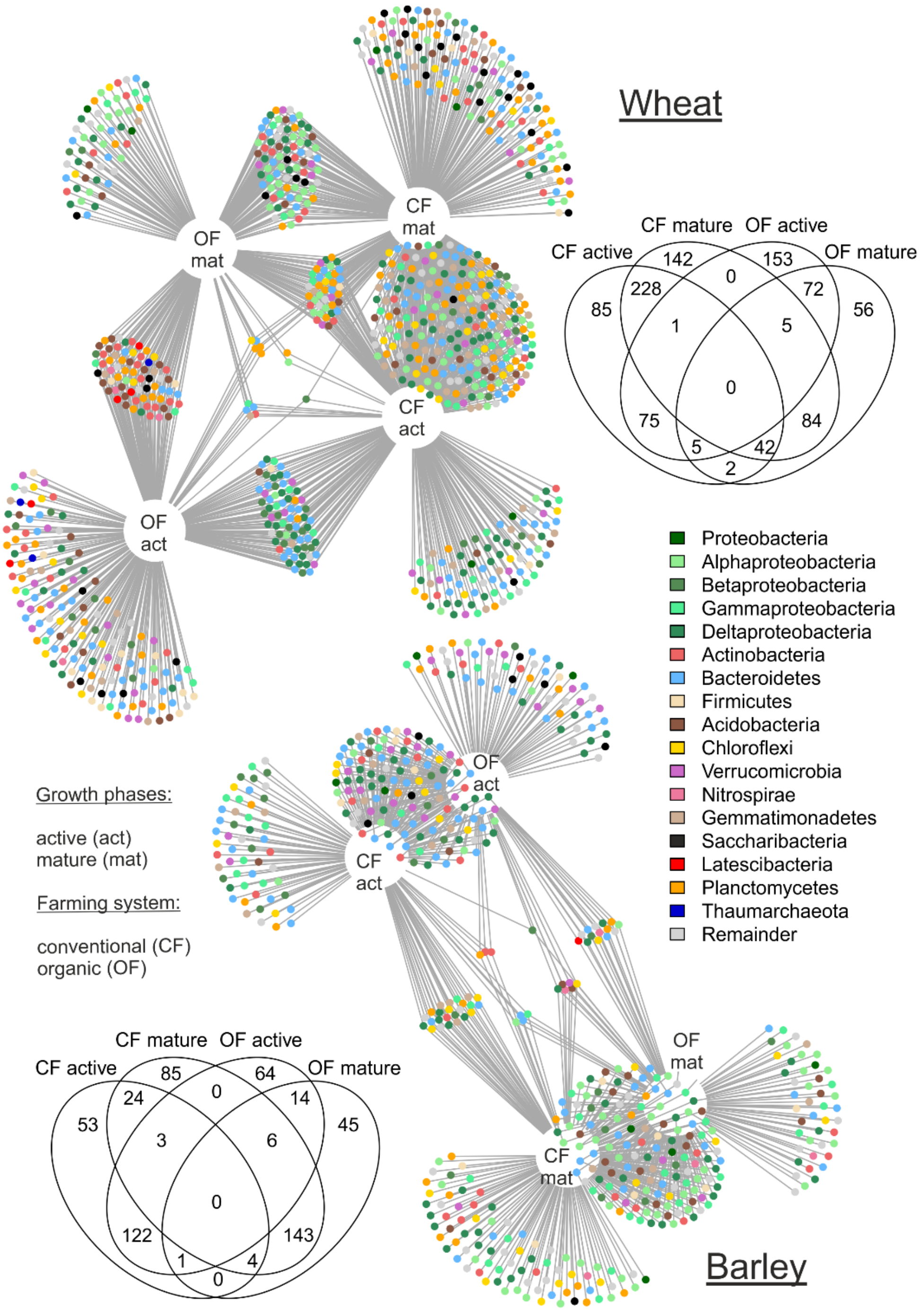

Figure SM 1 Indicator species analysis visualized as bipartite networks and Venn diagrams summarizing indicator species number and their phylogenetic assignment at different growth phases of wheat and barley in conventional and organic farming soils. Circles represent bacterial OTUs significantly associated with single or combined treatments according to consistent results of correlation and likelihood ratio tests (p<0.05). Circles are colored according to the phylum. Remainder comprises low abundant species (cumulative cut-off was set to 98 %).

Table SM 7 Distribution of indicator species among treatments, given at the phylum level for wheat. Enrichments in the occurrence of indicator species within treatments compared to overall community in

the rhizosphere of wheat (Total OTU) were estimated by Fisher test. Significant differences are given as different letters and marked by grey background.

Wheat		Indicator species (OTU)						
			Conventional Farming		Organic Farming		Shared	Shared
	Total OTU	Total	active	mature	active	mature	two	three
Acidobacteria	**7917**	55 ab	2 a	10 ab	7 a	3 a	31	2
Actinobacteria	**9965**	64 a	2 a	11 a	13 a	5 a	29	4
Alphaproteobacteria	**2916**	85 cdefg	4 abc	1 a	14 b-f	11 a	49	6
ARKDMS-49	**2**	1 a-m	1 abc	0 abcd	0 a-f	0 a	0	0
Armatimonadetes	**311**	14 cdehikl	1 abc	2 abcd	2 a-f	0 a	8	1
Bacteria_unclassified	**1167**	12 abj	1 abc	1 abc	0 abc	5 a	5	0
Bacteroidetes	**3155**	144 hikl	16 b	25 cd	21 bdef	7 a	66	9
Bathyarchaeota	**2**	0 a-m	0 abc	0 abcd	0 a-f	0 a	0	0
Betaproteobacteria	**5458**	39 ab	5 ac	8 ab	3 a	2 a	20	1
BJ-169	**11**	0 a-m	0 abc	0 abcd	0 a-f	0 a	0	0
BRC1	**40**	5 chkl	1 abc	1 abcd	3 d	0 a	0	0
Candidatus_Berkelbacteria	**81**	0 a-jm	0 abc	0 abcd	0 a-f	0 a	0	0
Chlamydiae	**105**	0 abd-gijm	0 abc	0 abcd	0 a-f	0 a	0	0
Chlorobi	**44**	7 k	0 abc	0 abcd	1 a-f	1 a	4	1
Chloroflexi	**2539**	40 jm	4 abc	9 abcd	4 abc	2 a	19	2
Cyanobacteria	**109**	11 kl	1 abc	0 abcd	4 de	1 a	5	0
Deferribacteres	**1**	0 a-m	0 abc	0 abcd	0 a-f	0 a	0	0
Deinococcus-Thermus	**17**	1 a-m	0 abc	0 abcd	0 a-f	0 a	1	0
Deltaproteobacteria	**2275**	118 hikl	17 b	12 bcd	12 b-f	9 a	65	3
Elusimicrobia	**130**	5 c-m	0 abc	3 bcd	0 a-f	0 a	2	0
Euryarchaeota	**32**	0 a-m	0 abc	0 abcd	0 a-f	0 a	0	0
FBP	**58**	2 a-m	0 abc	0 abcd	1 a-f	0 a	0	1
FCPU426	**4**	1 a-m	0 abc	1 abcd	0 a-f	0 a	0	0
Fibrobacteres	**91**	4 a-m	1 abc	1 abcd	1 a-f	0 a	1	0
Firmicutes	**705**	20 cdefghijm	1 abc	11 d	4 a-f	0 a	3	1
Fusobacteria	**2**	0 a-m	0 abc	0 abcd	0 a-f	0 a	0	0
GAL15	**4**	0 a-m	0 abc	0 abcd	0 a-f	0 a	0	0
Gammaproteobacteria	**2886**	53 dfjm	9 bc	6 abc	2 ac	3 a	29	4
Gemmatimonadetes	**1486**	54 ceghil	9 bc	8 abcd	4 abcf	1 a	30	2
Gracilibacteria	**13**	1 a-m	0 abc	0 abcd	0 a-f	0 a	1	0
Hydrogenedentes	**15**	2 a-m	0 abc	0 abcd	1 a-f	0 a	1	0
Ignavibacteriae	**1**	1 a-m	0 abc	1 abcd	0 a-f	0 a	0	0
JTB23	**1**	0 a-m	0 abc	0 abcd	0 a-f	0 a	0	0
Latescibacteria	**115**	5 c-m	0 abc	2 abcd	0 a-f	0 a	3	0
Lentisphaerae	**1**	0 a-m	0 abc	0 abcd	0 a-f	0 a	0	0
Microgenomates	**30**	0 a-m	0 abc	0 abcd	0 a-f	0 a	0	0
Nitrospirae	**545**	4 abjm	0 abc	1 abcd	0 abcf	0 a	3	0
Omnitrophica	**48**	0 a-m	0 abc	0 abcd	0 a-f	0 a	0	0
Parcubacteria	**173**	0 abfgjm	0 abc	0 abcd	0 a-f	0 a	0	0
Peregrinibacteria	**40**	0 a-m	0 abc	0 abcd	0 a-f	0 a	0	0
Planctomycetes	**2056**	112 hikl	3 abc	16 cd	22 def	3 a	55	13
Proteobacteria_unclassified	**193**	5 a-m	1 abc	0 abcd	2 a-f	2 a	0	0
RBG-1_(Zixibacteria)	**1**	0 a-m	0 abc	0 abcd	0 a-f	0 a	0	0
Saccharibacteria	**1307**	30 cdefgjm	1 abc	5 abcd	15 def	1 a	8	0
Spirochaetae	**3**	0 a-m	0 abc	0 abcd	0 a-f	0 a	0	0
SR1_(Absconditabacteria)	**11**	1 a-m	0 abc	0 abcd	0 a-f	0 a	1	0
Synergistetes	**1**	0 a-m	0 abc	0 abcd	0 a-f	0 a	0	0
Tectomicrobia	**53**	2 a-m	0 abc	0 abcd	0 a-f	0 a	2	0
Tenericutes	**15**	0 a-m	0 abc	0 abcd	0 a-f	0 a	0	0
Thaumarchaeota	**361**	3 abdefgjm	0 abc	2 abcd	0 abcef	0 a	1	0
TM6_(Dependentiae)	**121**	2 a-m	0 abc	1 abcd	0 a-f	0 a	1	0
Verrucomicrobia	**1811**	47 cdefgm	5 abc	15 cd	6 abcef	0 a	18	3
Woesearchaeota_(DHVEG-6)	**14**	0 a-m	0 abc	0 abcd	0 a-f	0 a	0	0
WS2	**20**	0 a-m	0 abc	0 abcd	0 a-f	0 a	0	0
WWE3	**8**	0 a-m	0 abc	0 abcd	0 a-f	0 a	0	0
**Total**	**48462**	**950**	**85**	**153**	**142**	**56**	**461**	**53**

Table SM 8 Distribution of indicator species among treatments, given at the phylum level for barley. Enrichments in occurrence of indicator species within treatments compared to overall community in the rhizosphere of barley (Total OTU) were estimated by Fisher test. Significant differences are given as letters and additionally marked by grey background.

Barley		Indicator species (OTU)						
			Conventional Farming		Organic Farming		Shared	Shared
	Total OTU	Total	active	mature	active	mature	two	three
Acidobacteria	**8463**	23 a	1 a	1 a	0 a	3 a	16	2
Actinobacteria	**9218**	35 a	3 ab	6 abc	4 ab	3 a	17	2
Alphaproteobacteria	**3085**	63 b-g	1 abc	14 de	2 abcd	10 a	35	1
ARKDMS-49	**2**	1 a-j	0 abc	0 a-f	1 bcd	0 a	0	0
Armatimonadetes	**346**	8 b-j	1 abc	0 a-f	3 bcd	2 a	2	0
Bacteria_unclassified	**1281**	20 bcefhi	1 abc	4 abcde	6 cd	2 a	7	0
Bacteroidetes	**2881**	93 dgj	11 c	5 abcd	14 c	5 a	56	2
Bathyarchaeota	**1**	0 a-j	0 abc	0 a-f	0 abcd	0	0	0
Betaproteobacteria	**5369**	26 a	9 abc	1 ab	1 ab	1 a	13	1
BJ-169	**12**	0 a-j	0 abc	0 a-f	0 abcd	0 a	0	0
BRC1	**43**	1 a-j	0 abc	1 a-f	0 abcd	0 a	0	0
Candidatus_Berkelbacteria	**111**	1 a-j	0 abc	0 a-f	0 abcd	1 a	0	0
Chlamydiae	**122**	0 abcdhi	0 abc	0 a-f	0 abcd	0 a	0	0
Chlorobi	**48**	3 b-hj	0 abc	3 ef	0 abcd	0 a	0	0
Chloroflexi	**2683**	30 bhi	3 abc	8 cd	0 abd	2 a	16	1
Cyanobacteria	**125**	11 j	1 abc	5 f	0 abcd	1 a	4	0
Deferribacteres	1	0 a-j	0 abc	0 a-f	0 abcd	0	0	0
Deinococcus-Thermus	**18**	0 a-j	0 abc	0 a-f	0 abcd	0 a	0	0
Deltaproteobacteria	**2723**	79 cdeg	2 abc	14 def	8 bcd	5 a	49	1
Diapherotrites	**2**	0 a-j	0 abc	0 a-f	0 abcd	0	0	0
Elusimicrobia	**137**	2 a-j	0 abc	0 a-f	0 abcd	0 a	2	0
Epsilonproteobacteria	**1**	0 a-j	0 abc	0 a-f	0 abcd	0	0	0
Euryarchaeota	**36**	0 a-j	0 abc	0 a-f	0 abcd	0 a	0	0
FBP	**59**	2 a-j	2 abc	0 a-f	0 abcd	0 a	0	0
FCPU426	**4**	1 a-j	0 abc	0 a-f	1 abcd	0 a	0	0
Fibrobacteres	**118**	3 a-j	0 abc	0 a-f	0 abcd	1 a	2	0
Firmicutes	**698**	12 b-i	0 abc	2 a-f	1 abcd	1 a	8	0
Fusobacteria	**2**	0 a-j	0 abc	0 a-f	0 abcd	0 a	0	0
GAL15	**6**	0 a-j	0 abc	0 a-f	0 abcd	0 a	0	0
Gammaproteobacteria	**3009**	46 bfhi	9 bc	7 bcd	3 abcd	4 a	22	1
Gemmatimonadetes	**1600**	20 bhi	4 abc	0 abcd	0 abcd	2 a	14	0
Gracilibacteria	**26**	1 a-j	0 abc	0 a-f	0 abcd	0 a	1	0
Hydrogenedentes	**16**	0 a-j	0 abc	0 a-f	0 abcd	0 a	0	0
Ignavibacteriae	**1**	0 a-j	0 abc	0 a-f	0 abcd	0 a	0	0
JTB23	**1**	0 a-j	0 abc	0 a-f	0 abcd	0 a	0	0
Latescibacteria	**126**	1 a-j	0 abc	0 a-f	0 abcd	0 a	1	0
Lentisphaerae	**1**	0 a-j	0 abc	0 a-f	0 abcd	0 a	0	0
Microgenomates	**36**	0 a-j	0 abc	0 a-f	0 abcd	0 a	0	0
Nitrospirae	**620**	5 abhi	0 abc	1 abcde	0 abcd	1 a	2	1
Omnitrophica	**65**	0 a-j	0 abc	0 a-f	0 abcd	0 a	0	0
Parcubacteria	**329**	2 abcdhi	0 abc	0 a-f	0 abcd	0 a	2	0
Peregrinibacteria	**72**	0 a-j	0 abc	0 a-f	0 abcd	0 a	0	0
Planctomycetes	**2165**	28 bhi	3 abc	9 de	6 bcd	0 a	9	1
Proteobacteria_unclassified	**242**	7 b-hj	0 abc	1 a-f	1 abcd	1 a	4	0
RBG-1_(Zixibacteria)	**1**	0 a-j	0 abc	0 a-f	0 abcd	0 a	0	0
Saccharibacteria	**1256**	8 ahi	0 abc	1 abcd	4 bcd	0 a	3	0
Spirochaetae	**5**	0 a-j	0 abc	0 a-f	0 abcd	0 a	0	0
SR1_(Absconditabacteria)	**16**	3 efgj	0 abc	1 a-f	1 abcd	0 a	1	0
Synergistetes	**1**	0 a-j	0 abc	0 a-f	0 abcd	0 a	0	0
Tectomicrobia	**59**	0 a-j	0 abc	0 a-f	0 abcd	0 a	0	0
Tenericutes	**10**	0 a-j	0 abc	0 a-f	0 abcd	0 a	0	0
Thaumarchaeota	**376**	0 ai	0 abc	0 abcde	0 abcd	0 a	0	0
TM6_(Dependentiae)	**138**	1 a-i	0 abc	0 a-f	0 abcd	0 a	1	0
Verrucomicrobia	**1935**	28 bfhi	2 abc	1 abcd	8 cd	0 a	16	1
Woesearchaeota_(DHVEG-6)	**22**	0 a-j	0 abc	0 a-f	0 abcd	0 a	0	0
WS2	**24**	0 a-j	0 abc	0 a-f	0 abcd	0 a	0	0
WWE3	**14**	0 a-j	0 abc	0 a-f	0 abcd	0 a	0	0
**Total**	**49747**	**564**	**53**	**85**	**64**	**45**	**303**	**14**
